# Supplementary material for: Continent-wide genomic analysis of the African buffalo (Syncerus caffer)
Source: Commun Biol. 2024 Jun 29;7:792. doi: 10.1038/s42003-024-06481-2 (PMC11217449; doi:10.1038/s42003-024-06481-2)

Supplementary Data 1. Genome assembly statistics.

Supplementary Figure 1. Mean read depth across the putative African buffalo-specific regions. Mean read depth was calculated for each of the 74,659 novel regions within the reference genome, for 46 of the population samples, using Mosdepth (v0.3.4) [75]. The distribution of average coverage values across the population samples, for each novel region, is shown. There are only 1494 novel regions with a mean read depth <1 and 419 regions with no reads mapped across these 46 samples.

Supplementary Data 2. Genes identified in the buffalo-specific sequence, with Ensembl transcript, gene and protein identifiers, and GO terms where relevant. Note that the list is greater than the 583 identified genes, as some genes appear in the list more than once due to having different transcripts.

Supplementary Data 3. Details of buffalo samples for which genome sequences were generated and included in this study, including sample identification, subspecies, country of origin, region of origin, whether sequences were retained in analysis following filtering steps (0.0625 relatedness, 0.2 missingness), the population group the sample was assigned to, and a latitude/longitude of a central point in the respective sampling area.

Supplementary Figure 2 Principal Component analysis pre- & post-downsampling (i.e. following sample removal post filtering steps; 0.0625 relatedness, 0.2 missingness), with data for components 1 and 2 illustrated, samples are coloured by population grouping.

Supplementary Figure 3. Admixture evaluation metrics (A) cross-validation error, (B) number of iterations to converge and (C) H', at different values of K calculated using 100 bootstraps of 100,000 variants each.

Supplementary Figure 4. Admixture analysis and EvalAdmix results for K=2-15.

Supplementary Figure 5. Relate-inferred inverse coalescence rates (effective population sizes) for each of the larger sub-groups to themselves (dashed lines) and each other (solid lines). For this comparison, due to the smaller sample sizes, all West African animals were collated into one group.

Supplementary Data 4. Pairwise F_{ST} values for the nine population groupings (*S. c brachyceros*, *S. c. nanus*, *S. c. aequinoctialis*, intermediate (putative hybrids between *S. c. nanus*, *S. c. aequinoctialis*), *S. c. caffer* Uganda, *S. c. caffe*r Kenya/Tanzania, *S. c. caffer* Mozambique, *S. c. caffer* Zimbabwe/Botswana and *S. c. caffer* South Africa), and geographic distance as measured to centred latitude/longitude measurement for each grouping.

Supplementary Data 5. Details of genes identified to be under selection in the African buffalo, whether the gene has been previously identified to be in a selection peak in either the cow or water buffalo, and whether the gene is related to immune response function. Genes are grouped by (a) detected in both XPEHH and PR analyses of African buffalo (dark green), (b) detected in either XPEHH or PR analyses of African buffalo, and in both metrics for water buffalo or cow analyses (medium green), (c) detected in either XPEHH or PR analyses of African buffalo, and in one of the metrics for water buffalo or cow analyses (light green), or (d) none of the above (no colour).

Supplementary Note 1. A methodological summary of the genome annotation process undertaken at ENSEMBL.

Supplementary Figure 6. Mapping rate by longitude of three randomly selected samples per country. No obvious mapping bias was observed among the West African samples when mapping to the reference genome obtained from an East African sample.

Supplementary Figure 1

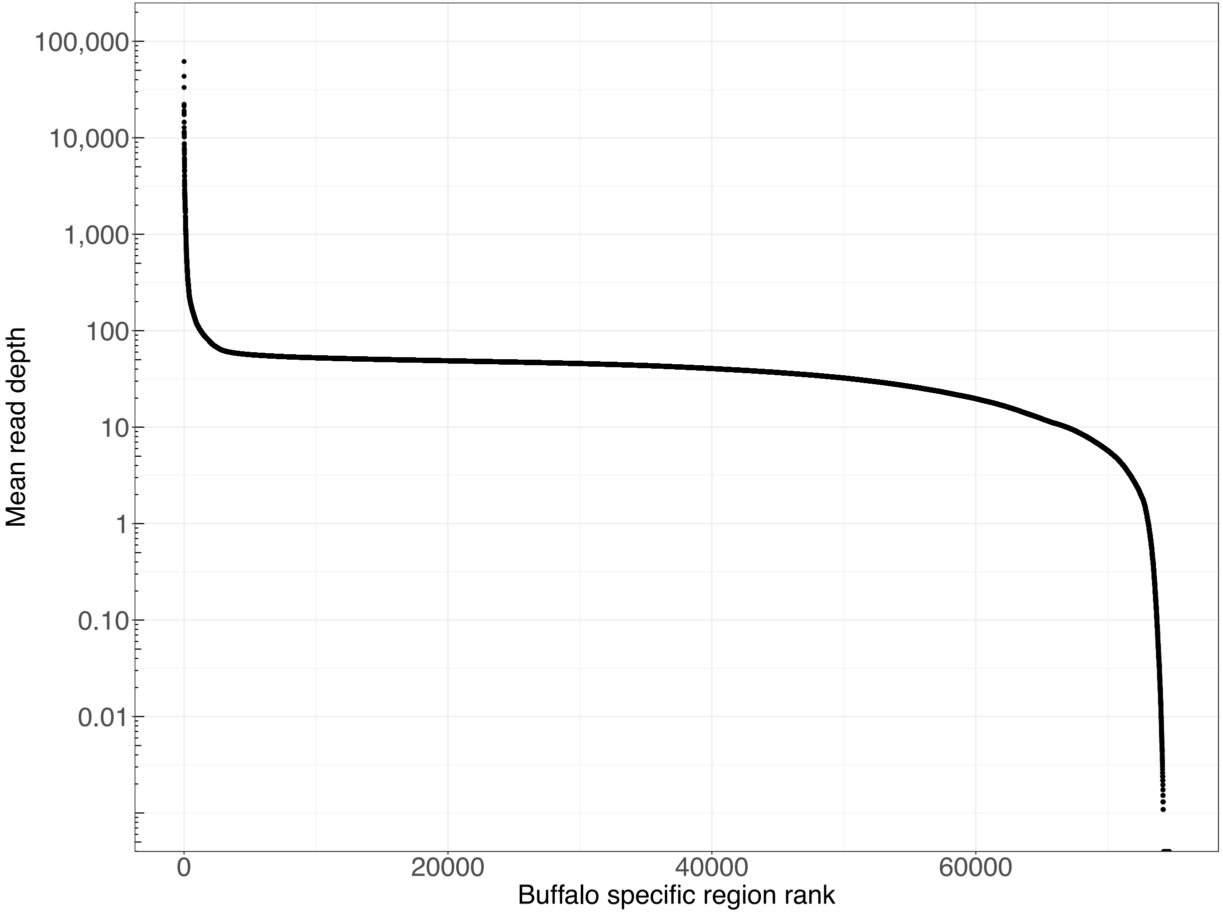

Supplementary Figure 2

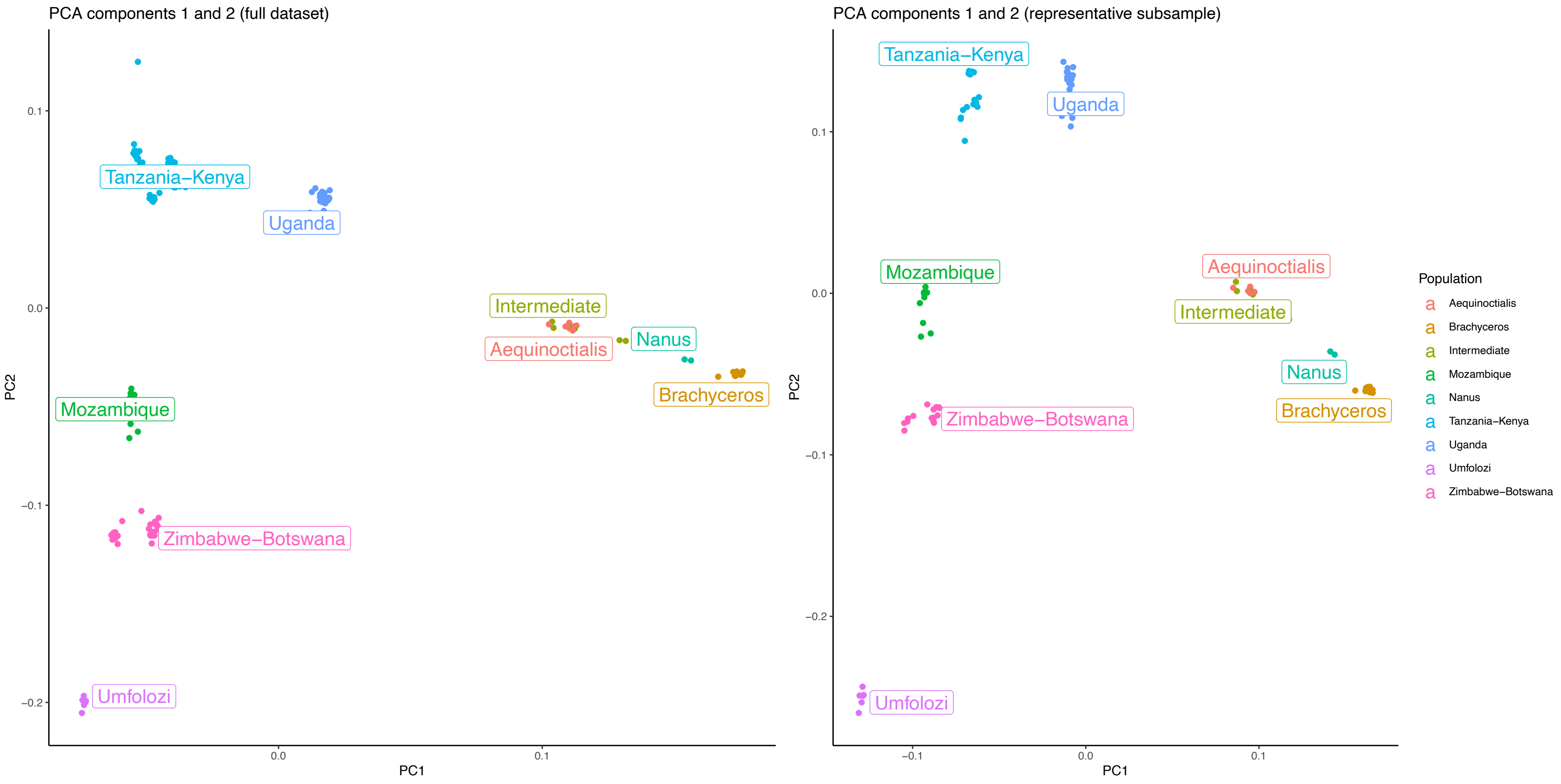

Supplementary Figure 3

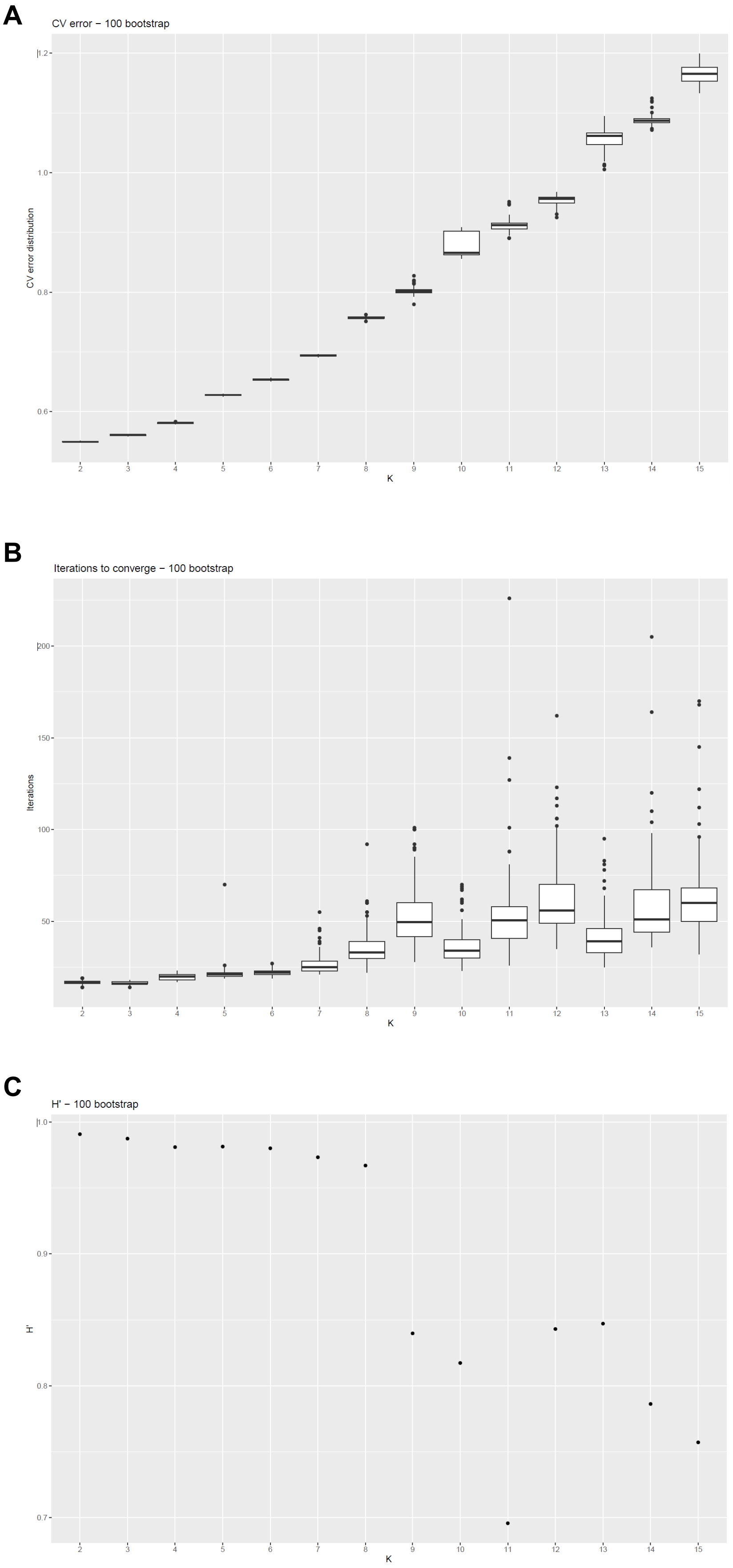

Supplementary Figure 4

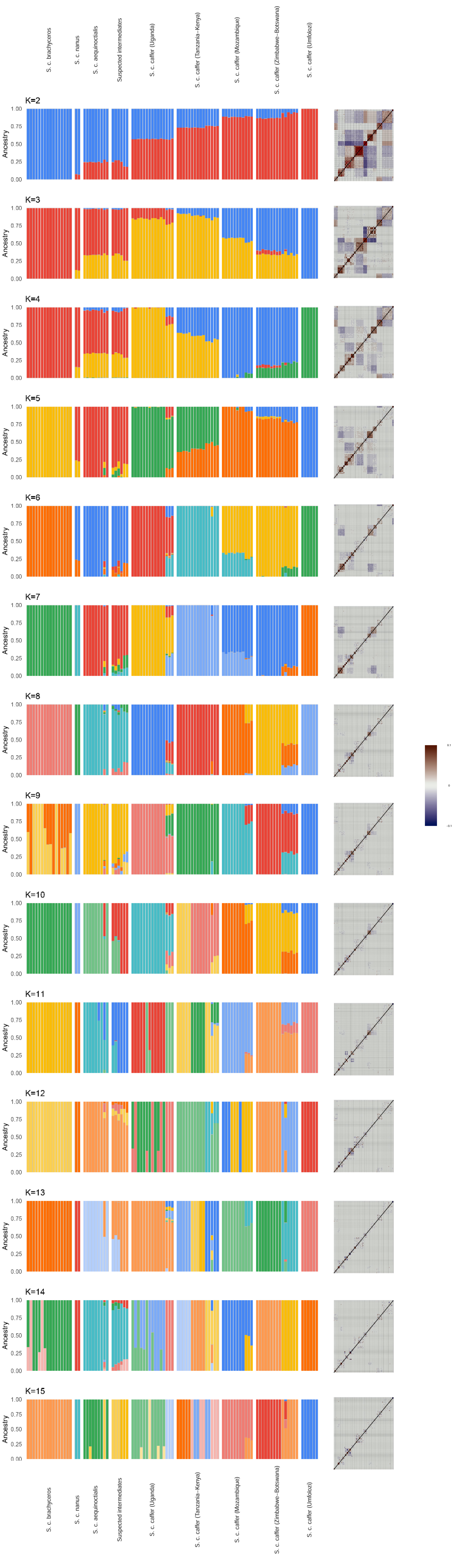

Supplementary Figure 5

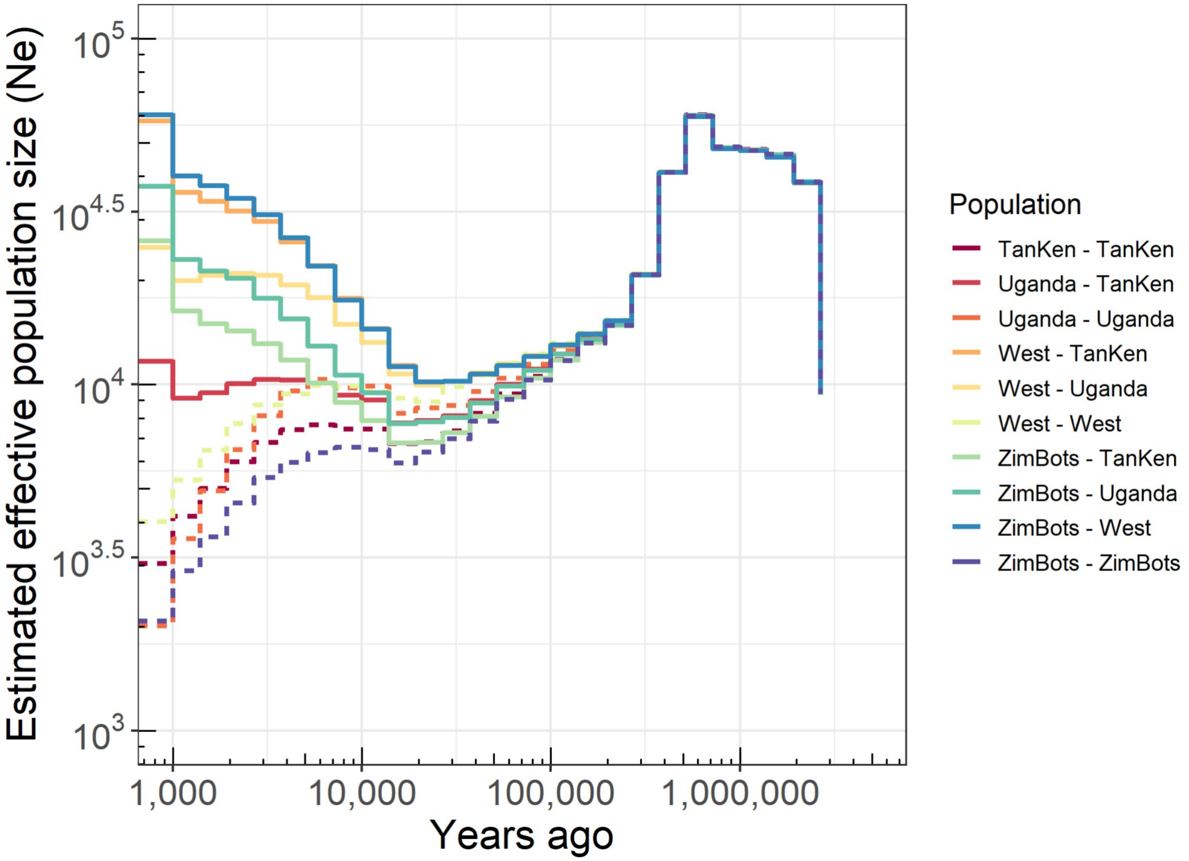

Supplementary Note 1

Ensembl Genome Annotation
Annotation of the assembly was created via the Ensembl gene annotation system (PMID: 27337980). A set of potential transcripts was generated using multiple techniques: primarily through alignment of transcriptomic data sets, and also through gap filling with protein-to-genome alignments of a sub-set of mammalian proteins from UniProt (PMID: PMC6323992). The UniProt mammalian proteins had experimental evidence for existence at the protein or transcript level (protein existence level 1 and 2). Additionally, a whole genome alignment was generated between the genome and the GRCh38 human reference genome using LastZ and the resulting alignment was used to map the coding regions of human genes from the GENCODE reference set.

At each locus, low quality transcript models were removed, and the data were collapsed and consolidated into a final gene model plus its associated non-redundant transcript set. When collapsing the data, priority was given to models derived from transcriptomic data. For each putative transcript, the coverage of the longest open reading frame was assessed in relation to known vertebrate proteins, to help differentiate between true isoforms and fragments. In loci where the transcriptomic data were fragmented or missing, homology data was used to gap fill if a more complete cross-species alignment was available, with preference given to longer transcripts that had strong intron support from the short-read data.

Gene models were classified, based on the alignment quality of their supporting evidence, into three main types: protein-coding, pseudogene, and long non-coding RNA. Models with hits to know proteins, and few structural abnormalities (i.e., they had canonical splice sites, introns passing a minimum size threshold, low level of repeat coverage) were classified as protein-coding. Models with hits to known protein, but having multiple issues in their underlying structure, were classified as pseudogenes. Single-exon models with a corresponding multi-exon copy elsewhere in the genome were classified as processed pseudogenes.

If a model failed to meet the criteria of any of the previously described categories, did not overlap a protein-coding gene, and had been constructed from transcriptomic data then it was considered as a potential lncRNA. Potential lncRNAs were filtered to remove transcripts that did not have at least two valid splice sites or cover 1000bp (to remove transcriptional noise).

A separate pipeline was run to annotation small non-coding genes. miRNAs were annotated via a BLAST (PMID: 2231712) of miRbase (PMID:30423142) against the genome, before passing the results in to RNAfold (PMID: 18424795). Poor quality and repeat-ridden alignments were discarded. Other types of small non-coding genes were annotated by scanning Rfam (PMID: 29112718) against the genome and passing the results into Infernal (PMID: 24008419).

The annotation for the African buffalo is available via Ensembl Rapid Release:
https://rapid.ensembl.org/Syncerus_caffer_GCA_902825105.1/Info/Index

Supplementary Figure 6

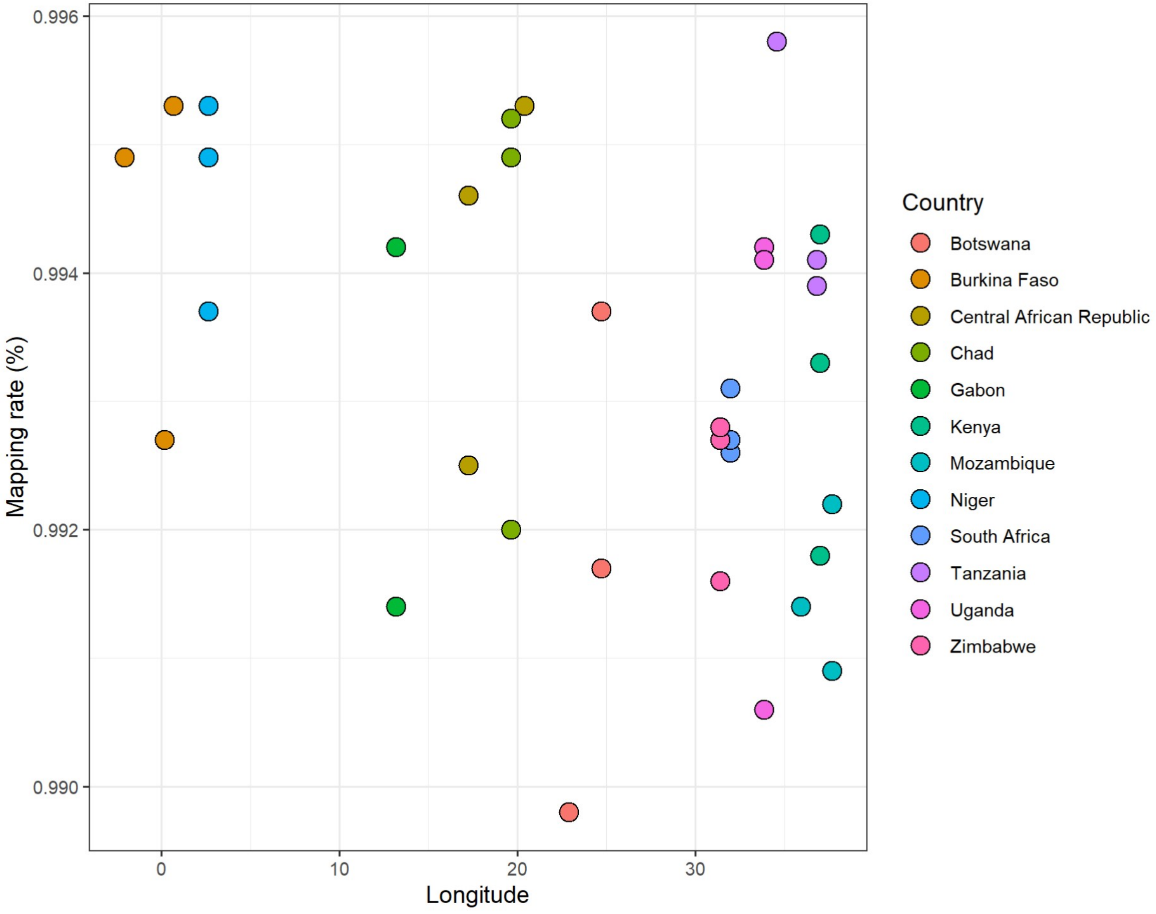

Supplement: Supplementary file 1 — Supplementary Information [file 42003_2024_6481_MOESM1_ESM.pdf]
