# Supplementary material for: Continent-wide genomic analysis of the African buffalo (Syncerus caffer)
Source: Commun Biol. 2024 Jun 29;7:792. doi: 10.1038/s42003-024-06481-2 (PMC11217449; doi:10.1038/s42003-024-06481-2)
Supplement: Supplementary file 2 — Description of Additional Supplementary Files [file 42003_2024_6481_MOESM2_ESM.pdf]

Description of Additional Supplementary Files

File name: Supplementary data 1
Description: Genome assembly statistics.

File name: Supplementary data 2
Description: Genes identified in the buffalo-specific sequence, with Ensembl transcript, gene and protein identifiers, and GO terms where relevant. Note that the list is greater than the 583 identified genes, as some genes appear in the list more than once due to having different transcripts.

File name: Supplementary data 3
Description: Details of buffalo samples for which genome sequences were generated and included in this study, including sample identification, subspecies, country of origin, region of origin, whether sequences were retained in analysis following filtering steps (0.0625 relatedness, 0.2 missingness), the population group the sample was assigned to, and a latitude/longitude of a central point in the respective sampling area.

File name: Supplementary data 4
Description: Pairwise FST values for the nine population groupings (S. c brachyceros, S. c. nanus, S. c. aequinoctialis, intermediate (putative hybrids between S. c. nanus, S. c. aequinoctialis), S. c. caffer Uganda, S. c. caffer Kenya/Tanzania, S. c. caffer Mozambique, S. c. caffer Zimbabwe/Botswana and S. c. caffer South Africa), and geographic distance as measured to centred latitude/longitude measurement for each grouping.

File name: Supplementary data 5
Description: Details of genes identified to be under selection in the African buffalo, whether the gene has been previously identified to be in a selection peak in either the cow or water buffalo, and whether the gene is related to immune response function. Genes are grouped by (a) detected in both XPEHH and PR analyses of African buffalo (dark green), (b) detected in either XPEHH or PR analyses of African buffalo, and in both metrics for water buffalo or cow analyses (medium green), (c) detected in either XPEHH or PR analyses of African buffalo, and in one of the metrics for water buffalo or cow analyses (light green), or (d) none of the above (no colour).
