# Supplementary material for: Continent-wide genomic analysis of the African buffalo (Syncerus caffer)
Source: Commun Biol. 2024 Jun 29;7:792. doi: 10.1038/s42003-024-06481-2 (PMC11217449; doi:10.1038/s42003-024-06481-2)
Supplement: Supplementary file 3 — Supplementary Data 1 [file 42003_2024_6481_MOESM3_ESM.pdf]

Supplementary Data 1a. Assembly statistics at each round of polishing.

Assembly	Unfilled	Filled	pilon1	pilon2	pilon3	pilon4
# sequences (>= 0 bp)	3,351	3,351	3,351	3,351	3,351	3,351
# sequences (>= 1000 bp)	3,041	3,042	3,043	3,043	3,043	3,043
# sequences (>= 5000 bp)	2,136	2,157	2,156	2,155	2,154	2,154
# sequences (>= 10000 bp)	1,352	1,378	1,379	1,380	1,380	1,381
# sequences (>= 25000 bp)	320	325	325	325	325	325
# sequences (>= 50000 bp)	208	208	209	209	211	211
Total length	2,651,354,003	2,651,819,263	2,652,421,646	2,652,507,212	2,652,733,160	2,652,945,513
GC (%)	41.76	41.76	41.75	41.75	41.75	41.75
Scaffold N50	69,170,419	69,170,419	69,160,108	69,159,231	69,159,740	69,160,875
N75	31,468,510	31,468,510	31,463,761	31,459,774	31,459,818	31,459,638
L50	13	13	13	13	13	13
L75	27	27	27	27	27	27
# N's per 100 kbp	149	149	123	116	113	112

Supplementary Data 1b. QUAST Assembly metrics. Contig length distribution, and N50 metrics for the genome assembly prior and post PBJelly gap filling and after each successive round of polishing with Pilon.

Assembly	Unfilled	Filled	Pilon1	Pilon2	Pilon3	Pilon4
# contigs (>= 0 bp)	3,351	3,351	3,351	3,351	3,351	3,351
# contigs (>= 1000 bp)	3,041	3,042	3,043	3,043	3,043	3,043
# contigs (>= 5000 bp)	2,136	2,157	2,156	2,155	2,154	2,154
# contigs (>= 10000 bp)	1,352	1,378	1,379	1,380	1,380	1,381
# contigs (>= 25000 bp)	320	325	325	325	325	325
# contigs (>= 50000 bp)	208	208	209	209	211	211
Largest contig	193,316,640	193,316,640	193,289,354	193,281,852	193,285,335	193,288,002
Total length	2,651,354,003	2,651,819,263	2,652,421,646	2,652,507,212	2,652,733,160	2,652,945,513
N50	69,170,419	69,170,419	69,160,108	69,159,231	69,159,740	69,160,875

Supplementary Data 1c. QUAST Assembly metrics compared against *Bubalus bubalis* reference genome. Genome length, alignment and assembly metrics for the genome assembly prior and post PBJelly gap filling and after each successive round of polishing with Pilon.

Assembly	Unfilled	Filled	Pilon1	Pilon2	Pilon3	Pilon4
Reference length	2.65 Gb	2.65 Gb	2.65 Gb	2.65 Gb	2.65 Gb	2.65 Gb
GC (%)	41.76	41.76	41.75	41.75	41.75	41.75
Reference GC (%)	41.81	41.81	41.81	41.81	41.81	41.81
# misassemblies	10,283	10,289	55,205	55,190	55,180	55,219
# misassembled contigs	594	592	1,024	1,021	1,027	1,028
# unaligned contigs	357 + 1377 part	358 + 1413 part	265 + 823 part	265 + 834 part	266 + 829 part	261 + 833 part
Unaligned length	445,613,097	446,253,486	73,191,862	73,130,730	73,194,004	73,190,626
Genome fraction (%)	82	82	94	94	94	94
# N's per 100 kbp	149	149	123	116	113	112
# mismatches per 100 kbp	4,324	4,325	2,104	2,104	2,104	2,104
# indels per 100 kbp	202	202	258	256	256	255
Largest alignment	6,016,346	6,016,343	1,669,457	1,669,468	1,669,432	1,669,453
Total aligned length	2.2 Gb	2.2 Gb	2.571 Gb	2.571 Gb	2.572 Gb	2.572 Gb
NA50	674,686	674,686	128,221	128,284	128,283	128,332
NGA50	673,117	673,117	127,916	128,119	128,113	128,156

Supplementary Data 1d. Genome Assembly Scaffold Metrics. N, L, NG, LG and GC content for a given proportion of the genome assembly, x.

x	Nx	Lx	NGx	LGx	GC%
5	193,288,002	0	193,288,002	0	40.17
10	143,084,846	1	143,084,846	1	40.99
15	136,240,748	2	136,240,748	2	40.87
20	111,633,540	3	111,633,540	3	41.03
25	104,800,038	4	104,800,038	4	40.87
30	94,608,040	6	94,608,040	6	40.6
35	89,027,109	7	89,027,109	7	40.6
40	73,376,136	9	73,376,136	9	40.72
45	71,999,969	10	71,869,821	11	40.73
50	69,160,875	12	66,444,531	13	40.82
55	63,542,860	14	63,539,877	15	41.17
60	56,889,046	16	55,993,668	17	41.38
65	47,354,412	19	43,700,853	20	41.33
70	39,306,693	22	38,815,046	23	41.33
75	31,459,638	26	26,790,382	27	41.46
80	25,550,784	31	23,490,963	32	41.62
85	19,164,195	37	15,575,171	39	41.65
90	8,757,406	46	6,900,340	52	41.65
95	3,798,400	68	1,747,249	84	41.73
100	2	3,351	2	3351	41.7
